# Treatment default and its related factors among tuberculosis patients, a case-control study in Iran

**DOI:** 10.3205/dgkh000368

**Published:** 2020-12-10

**Authors:** Mahdi Afshari, Mohsen Aarabi, Mohammadreza Parsaee, Asghar Nezammahalleh, Mahmood Moosazadeh

**Affiliations:** 1 Department of Community Medicine, School of Medicine, Zabol University of Medical Sciences, Zabol, Iran; 2 Department of Family Medicine, School of Medicine, Mazandaran University of Medical Sciences, Sari, Iran; 3 Health deputy, Mazandaran University of Medical Sciences, Sari, Iran; 4 Gastrointestinal Cancer Research Center, Non-communicable Diseases Institute, Mazandaran University of Medical Sciences, Sari, Iran

**Keywords:** tuberculosis, treatment default, risk factors, mycobacterium, infection

## Abstract

**Introduction:** Treatment default is one of the main challenges in tuberculosis (TB) control and is considered a major barrier to achieving the sustainable development goals (SDG). Identifying the factors associated with this outcome can help us provide appropriate strategies for decision making. This study investigates the determining factors of treatment default among TB patients.

**Methodology:** In this case-control study, all 88 TB patients experiencing treatment default during an11-year-period in Mazandaran province, Iran, were compared with 176 randomly selected TB patients without a history of default. Cases and controls were matched based on the year of incidence as well as the treatment center. Related factors of treatment default were determined using multivariate logistic regression models.

**Results:** For men, the odds ratio of experiencing treatment default was 1.67 (p=0.165). In addition, considering ages >64 years as the reference group, the odds ratios for 15- to 24- and 55- to 64-year-olds were 0.95 (p=0.940) and 0.37 (p=0.123), respectively. The corresponding odds ratios for patients 25–34, 35–44 and 45–54 years of age were 1.29 (p=0.547), 1.40 (p=0.472), and 1.39 (p=0.512) respectively. Moreover, the odds ratios for urban residents, patients with a history of imprisonment, a history of previous treatment, adverse treatment effects, previous exposure, non-Iranians and patients with smear-positive TB were 1.72 (p=0.070), 1.24 (p=0.657), 1.47 (p=0.756), 0.99 (p=0.998), 0.98 (p=0.960), 9.29 (p=0.010), and 2.27 (p=0.049) respectively.

**Conclusion:** Non-Iranian nationality and smear-positive TB were detected as predictors of treatment default among patients with tuberculosis.

## Zusammenfassung

**Einleitung:** Therapieversager sind eine der größten Herausforderungen bei der Bekämpfung der Tuberkulose (TB) und eine wesentliche Barriere für das Erreichen des Ziels einer nachhaltigen Entwicklung. Die Identifizierung von für die Behandlung wesentlichen Faktoren hilft, Strategien zur Entscheidungsfindung zu entwickeln. Ziel dieser Studie ist es, die bestimmenden Faktoren für Therapieversagen bei Tuberkulosepatienten zu untersuchen.

**Methode:** In der Fall-Kontroll-Studie wurden 88 TB-Patienten, die während eines Zeitraums von 11 Jahren in der Provinz Mazandaran, Iran, Therapieversager erlitten, mit 176 zufällig ausgewählten TB-Patienten ohne Vorgeschichte mit Therapieversagen verglichen. Die Fälle und Kontrollen wurden auf der Grundlage des Jahrs des Auftretens sowie des Behandlungszentrums gematcht. Relevante Faktoren des Therapieversagens wurden mit Hilfe multivariater logistischer Regressionsmodelle untersucht.

**Ergebnisse:** Die Odds Ratio für Therapieversager betrug bei Männern 1,67 (p=0,165). Betrachtet man darüber hinaus das Alter über 64 Jahre als Referenzgruppe, betrug die Odds Ratio für die Altersgruppe 15-24 0.95(p=0.940), für die Altersgruppe 25–34 1.29 (p=0.547), für die Altersgruppe 35–44 1.40 (p=0.472), für die Altersgruppe 45–54 1.39 (p=0.512) und für die Altersgruppe 55–64 Jahre 0,37 (p=0,123). Die Odds Ratios betrugen für Stadtbewohner, Personen mit einer Vorgeschichte von Inhaftierungen, Personen mit einer Vorgeschichte früherer Behandlungen mit Nebenwirkungen, Personen mit früherer Exposition, Nicht-Iraner und Personen mit positivem TB-Ausstrich 1,72 (p=0,070), 1,24 (p=0,657), 1,47 (p=0,756), 0,99 (p=0,998), 0,98 (p=0,960), 9,29 (p=0,010) bzw. 2,27(p=0,049).

**Schlussfolgerung:** Nur Nicht-iranische Staatsangehörigkeit und Personen mit positivem TB Ausstrich erwiesen sich als Prädiktoren für Therapieversager bei Tuberkulosepatienten.

## Introduction

Tuberculosis (TB) is an infectious necrotizing disease caused by *Mycobacterium tuberculosis* which leads to acute or chronic involvement of all organs [1], [2]. TB cases are the main source of infection spread in the community. Early diagnosis and treatment of these cases is the most effective control strategy of the disease [3], [4]. Without effective treatment, a smear-positive TB patient can infect 15–20 healthy individuals per year. Delayed diagnosis, HIV/AIDS, immunosuppressive disorders and multi-drug resistant TB (MDR) are the greatest challenges to controlling this disease [3].

The World Health Organization has defined treatment default as missing cases during follow-up, including TB patients who did not begin treatment or interrupted the treatment for at least two months [5]. Such interruption has been reported for different reasons, such as adverse drug effects, ignorance, replacement of standard anti-TB treatment with herbal medicine, and patients’ non-compliance due to stigmatization. These patients can weaken the TB control program because they are at higher risk of clinical deterioration, complications, relapse, failure, resistance development and death. Moreover, the infection can persist in these cases and act as a permanent source of infection, spreading within the community [6], [7], [8], [9]. 

In 2015, the treatment success rates for TB patients globally, in the eastern Mediterranean region, and Iran have been reported as 83%, 91%, and 87%, respectively [10]. Of 6,489 TB cases followed-up from 2005–2011 at Tehran University of Medical Sciences, Iran, treatment default among smear-positive cases, smear-negative cases, unknown cases, extra-pulmonary cases, and those with a history of previous treatment was 4.5%, 4%, 3.4%, 2.9%, and 9.4% respectively [11]. The treatment default rate in Sudan [12] and Nigeria has been reported as 8.1% and 9.9%, respectively. In Nigeria, such non-adherence was more common among older persons, those with smear-negative and extra-pulmonary TB, as well as those with long-term treatment [9].

The rates described above indicate that treatment default is a major challenge to TB control programs and the main obstacle for achieving sustainable development goals [10]. Detecting the factors associated with treatment default can help policy makers provide effective strategies for TB control. Such factors can vary even in different parts of one country. According to our knowledge, there is no data on the determinants of treatment default in the northern parts of Iran. Therefore, we aimed to investigate the role of some of the most important factors in the treatment default of TB patients in this region.

## Materials and methods

This case-control study was carried out among a cohort of TB patients in Mazandaran-northern province in Iran. Cases were patients with history of treatment default according to the WHO criteria [5]. Controls were TB cases whose treatment had been completed without interruption. The study protocol was approved by the institutional ethics committee of Zabol University of Medical Sciences (ethics code: zbmu.1.REC.1396.221). All required ethical issues were respected in accordance with the Helsinki declaration. 

All TB cases registered from 2005 to 2017 in the study area and who experienced anti-TB treatment interruption (88 cases; 14 females, 74 males) were selected as the case group. It should be noted that treatment default was defined as interruption of an anti-TB treatment for two months or more following at least one month of anti-TB treatment [7], [9]. Controls were randomly selected of TB patients with continuous treatment, i.e., without a history of treatment default. Two controls were selected per case, and matched for the year of disease onset as well as the health centersfor managing and monitoring the treatment. The source of data was the TB register software program at Mazandaran University of Medical Sciences. In the case of missing data, the patients or their health supervisor would be visited by the researchers and required information was provided. The variables extracted from the software program – including gender, age, area of residence (urban/rural), history of imprisonment, close contact with TB patients, previous treatment, type of TB, treatment complications, HIV infection, and nationality – were entered into excel format and transferred to STATA version 14 software for descriptive statistics and statistical analysis. 

The data were described as percent frequency, mean, standard deviation, minimum and maximum. The investigated factors were compared between cases and controls using the Chi-squared or Fisher’s exact tests (categorical variables) or independent T-/Mann-Whitney tests (continuous variables). The predicting variables for treatment default were investigated using multivariate logistic regression models. Statistical significance was set at p<0.05. The efficacy of the logistic models was assessed using Nagelkerke’s R^2^. 

## Results

Among 3,715 TB patients registered from 2005 to 2017, 2.37% had treatment default. Frequencies of treatment default among smear-positive, smear-negative and extra-pulmonary TB patients were 3.16% (62/1,964), 1.98% (14/705) and 1.15% (12/1,046), respectively.

A total of 264 TB patients were investigated in this study (88 cases; 14 females, 74 males) and 176 controls; 55 females, 121 males). Mean (SD) age of cases and controls was 42.4 (18.5) and 46.5 (18.6) years, respectively (p=0.094). 

The frequencies of males (p=0.007), urban residents (p=0.094), non-Iranians (p<0.001), smear-positive TB cases (p=0.036), 15- to 34-year-olds (p=0.167), patients with a history of imprisonment (p=0.364) and unknown HIV status (p=0.227) were higher among cases than controls. Moreover, adverse drug effects (p=0.632) and close contact with TB cases (p=0.666) were more common among controls than cases (Table 1 (Tab. 1)). Only the differences of gender, nationality and TB type were statistically significant between cases and controls. 

Multivariate logistic regression models showed that the odds ratios for non-Iranian nationality (9.29, 95% confidence interval: 1.69–50.90) and smear-positive TB (2.27, 95% confidence interval: 1–5.15) were statistically significant (Table 2 (Tab. 2)). The variables entered into the final model predicted 17.6% of the dependent variable.

## Discussion

In this study, the odds of default during TB treatment was 67% higher among men than women. It was also 29%, 40%, and 39% higher in 25- to 34-year-olds, 35- to 44-year-olds and 45- to 54-year-olds, respectively. However, the odds of treatment default was5% and 63% lower among age groups 15–24 and 55–64 years, respectively. In addition, urban residents, patients with a history of imprisonment, non-Iranians, patients undergoing re-treatment, smear-positive, and smear-negative TB patients had 72%, 24%, 9.3-fold, 47%, 2.3-fold and 1.3-fold higher chance of treatment default, respectively. Moreover, patients experiencing adverse drug reactions during treatment as well as those with a history of close contact had a 1% and 21% lower chance of treatment default, respectively. 

Based on the results of a study conducted in Nigeria, risk of treatment default increased with age, negative sputum smear before treatment, in extra-pulmonary TB patients and patients receiving long-term treatment [9]. Only long-term treatment (in patients receiving re-treatment) was associated with treatment default in our study.

In a study conducted by Sengul et al. [13] in Turkey, older ages, a history of previous anti-TB treatment, co-morbidities, drug resistance and low educational level were found to be risk factors of the adverse outcomes among TB patients. It should be noted that in the Sengul study, different treatment outcomes were investigated. 

Ignorance (16.7%), traveling to a distant area (12.5%), feeling well (11.7%) and adverse drug reactions (10.8%) have been reported as some of the factors responsible for treatment default in Kenya. In that study, logistic regression models showed little knowledge, the use of herbal medicine, HIV co-morbidity and male gender to be independent predictors of treatment default [7].

Another study carried out in South Africa reported that the chance of treatment default was higher among males, patients with previous TB treatment, those aged 15–24, and unknown HIV status [14]. 

In contrast, in Brazil, treatment default was more common among TB/HIV patients and those aged 15–39 years (compared to younger patients) [15], while in Sudan, rural residents, patients experiencing treatment side-effects, and those with a history of tuberculosis defaulted their treatment more often than did the other TB patients [16]. Similarly, in Tajikistan, adverse drug reactions and previous anti-TB treatment were predictors of treatment default [17]. 

However, two studies carried out in Brazil [18] and India [19] identified drug and alcohol abuse as independent risk factors of treatment default. The latter study reported adverse drug effects as well as long distance from treatment centers and little knowledge about TB as other reasons for treatment default.

All the above evidence agreed well regarding the role of male gender and history of previous anti-TB treatment in the increasing the probability of treatment default. Conversely, we observed higher rates of treatment default among urban residents. It should be noted that the coordination and cooperation between TB patients and health staff are better in the rural regions of the study area, and there is better access to health services. Although different age groups were responsible for increasing the risk of treatment default, there is more agreement between the studies regarding the 14- to 44-year age group. In contrast to the other studies, the current study found a higher rate of default among smear-positive TB patients compared to those with extra-pulmonary TB. Investigating the reasons for default in these patients in future studies is suggested. In addition, although the number of non-Iranian patients was low, they showed more non-compliance during treatment, which can be pose considerable health challenges for the countries of origin and destination. 

Some of the studies mentioned several reasons for treatment default among TB patients, and their results can be helpful. However, it is better to conduct independent and comprehensive research in this regard. According to a systematic review, these reasons were economic, social, transportation-related, but poor relationships between patients and health staff also played a role [20]. A qualitative study reported inappropriate nutrition, belief in traditional medicine, poor communication with health providers, and lack of access to the health services, discrimination and stigmatization as causes of poor compliance in Ethiopia [21]. The corresponding reasons in Rio de Janeiro, Brazil were reported by Maciel et al. [22] as economic condition, quality of the TB surveillance system and also infrastructure.

One of the limitations of the current study is that none of the patients who defaulted treatment were HIV positive, and most had an unknown HIV status. Moreover, just one of the patients with treatment default had a previous history of anti-TB treatment. 

In conclusion, our study revealed that non-Iranian nationality and smear-positive TB are predictors of treatment default. However, living in urban areas showed a borderline (with P-value between 0.05 and 1) relationship. To control these adverse outcomes and improve the quality of TB control programs, an exact and comprehensive list of non-Iranian patients as well as their immigration status should be provided and permanently monitored. In addition, cooperation between generations should be considered for their systematic follow-up during treatment. The observed non-compliance of smear-positive TB patients can increase the spread of infection, leading to major and irreversible damage to the TB control program. To facilitate achieving the sustainable development goals [10], all factors associated with treatment default of patients should be identified during further qualitative and quantitative studies. 

## Notes

### Competing interests

The authors declare that they have no competing interests.

### Acknowledgment

The authors thank Zabol University of Medical Sciences for the kind financial support and Mazandaran University of Medical Sciences for providing the required data.

## Figures and Tables

**Table 1:**
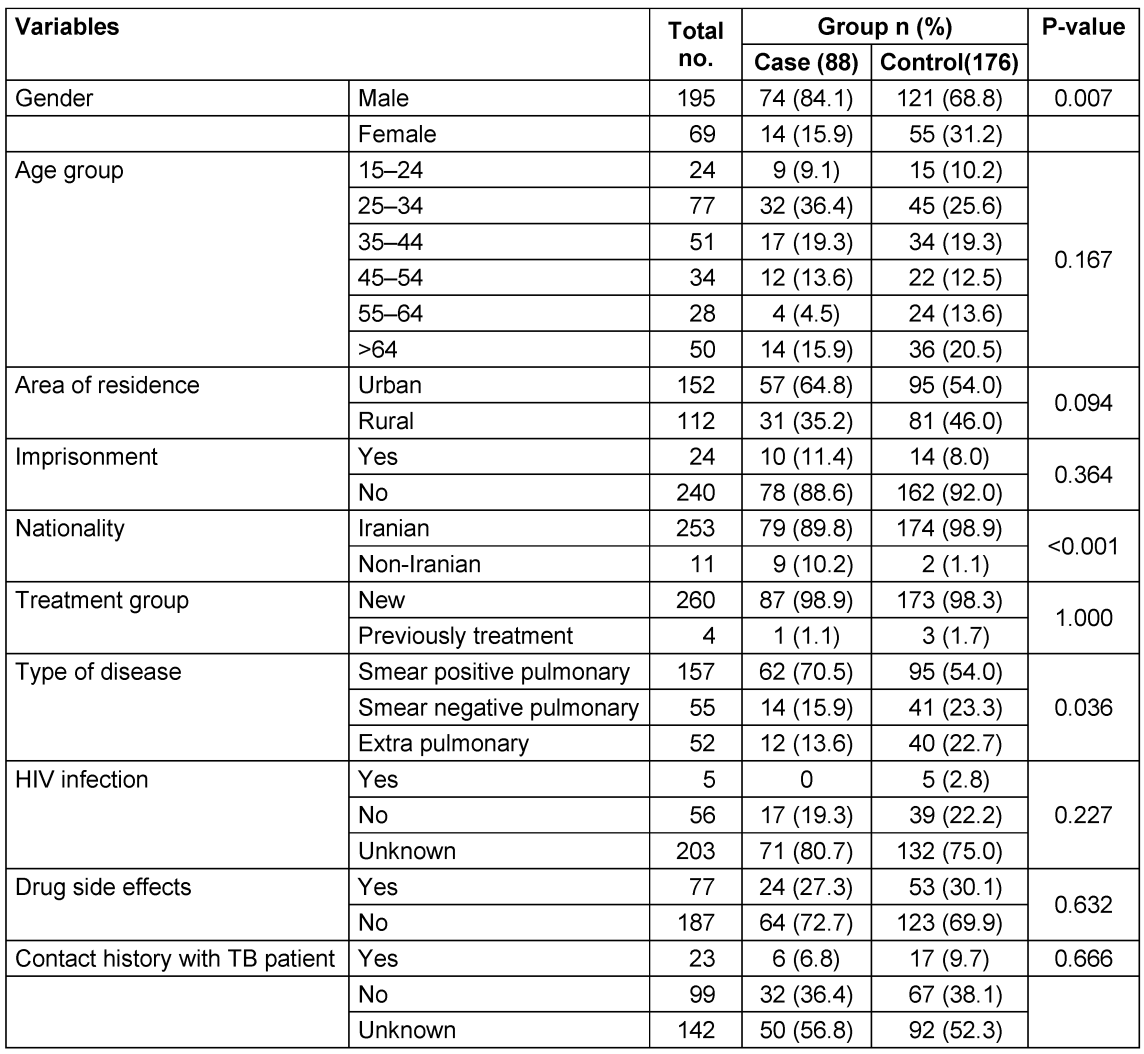
Epidemiological characteristics of the cases and controls

**Table 2:**
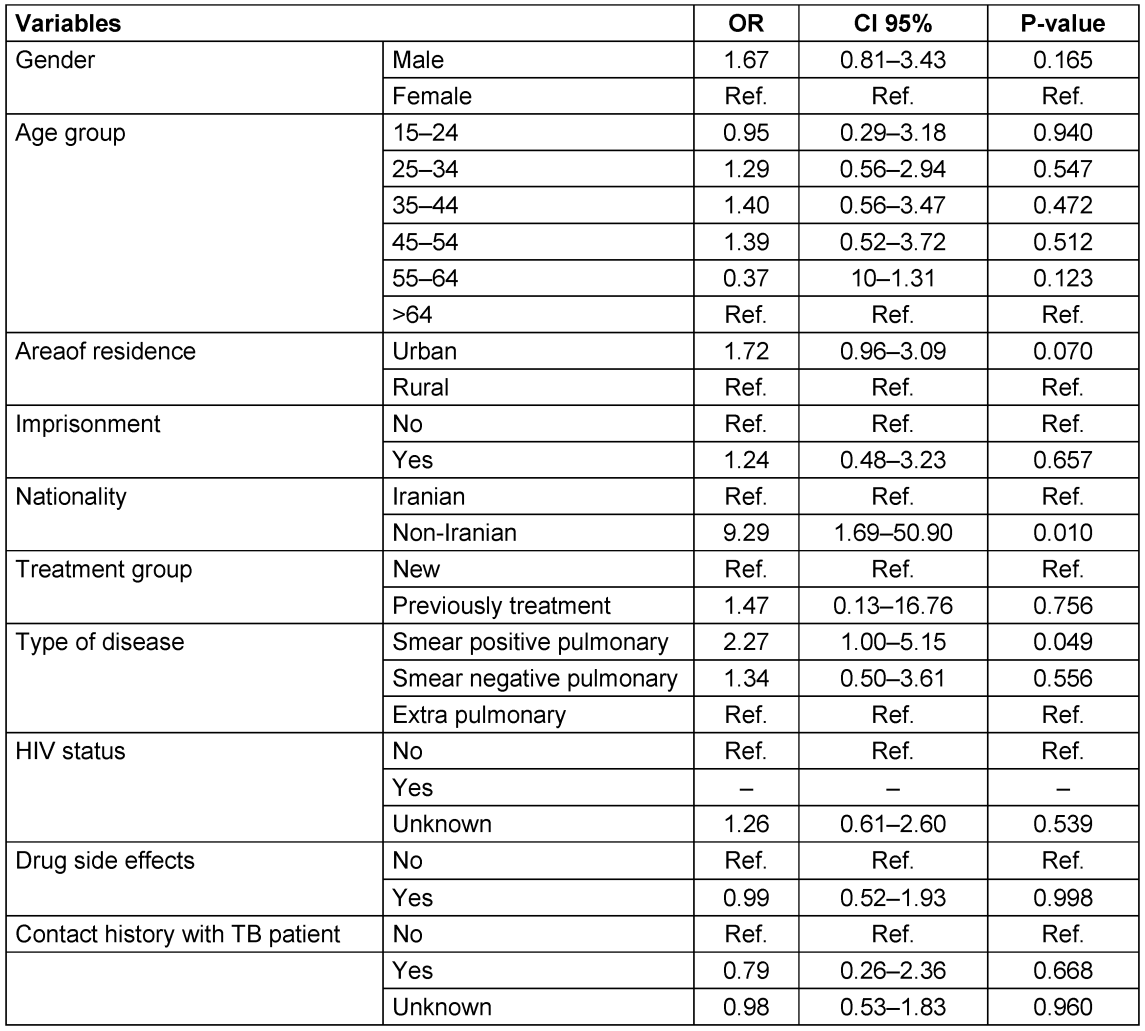
Factors associated with treatment default
